# Emotional intelligence, distancing, and learning a new skill as strategies to combat the deleterious effects of emotional labor on attorney wellbeing

**DOI:** 10.3389/fnbeh.2023.1221145

**Published:** 2023-07-26

**Authors:** Norina Melita

**Affiliations:** New York State Unified Court System, New York, NY, United States

**Keywords:** emotional intelligence, emotional labor, cognitive dissonance, distancing, attorney wellbeing

## Introduction

The idea for this article came to me while I was in a deposition on a large property damage case. At the time of the deposition, the case was already 5 years old. There had been a previous motion to compel or strike against my client for failure to produce some documents, which eventually was decided in my client's favor. The Order had a clause by which my client would be sanctioned if it was later found that documents it said it did not possess or did not exist, were later discovered. During the deposition, opposing counsel was attempting to ask my client's representative about the same documents that had been the subject of her prior motion. The deposition was getting heated. I could feel my blood pressure rising as I continued to object to her questions. I finally objected and said that the line of questioning was either asked and answered or addressed by the Court in its previous order. Opposing counsel finished by saying “We'll see” and moved on to other questions.

When we took a break, I could still feel the blood pumping through my veins and my head spinning. We ate lunch in the same room the deposition was being held. Everyone was hungry. During lunch, opposing counsel and I, along with the other counsel and clients in the room, casually exchanged pointers about Adirondack lakes in the area, discussed the lake houses we owned on various bodies of water, and generally made small talk. I could not help but think during this time how contrived it all felt, knowing that we had, only minutes ago, fought on the record. Back on the record, our “game faces” were restored as we returned from lunch.

I never forgot that exchanged and it was the impetus for writing this article. Trying to name this odd feeling I had felt that day, I kept thinking it was some sort of “emotional incongruity.” But I knew that was not quite the word I was looking for. Once I started doing my research, I came across “emotional dissonance” and that struck a chord. I kept reading and slowly fell down a rabbit hole. My journey brought me to this article, through a wide, meandering tunnel of subjects like emotional labor, emotional intelligence, neuroplasticity, meditation, and psycholinguistics. Throughout the process, introspection abounded.

In this article I attempt to summarize the neurobiological and physiological effects of emotional labor on attorneys, as well as provide several means of counteracting them. In particular, the role of emotional intelligence and mindfulness in improving attorney wellbeing is discussed. Concepts of reappraisal and linguistic distancing present in journaling are also discussed. Finally, the benefits of taking up a new skill on neurological, psychological, and physiological health are discussed. It is my hope that the extensive research discussed in this article can be used by Bar Associations to educate attorneys in the hazards of emotional labor and design programs to facilitate attorney wellbeing.

While there may be a number of other ways to combat the deleterious effects that emotional labor has on an attorney's wellbeing, the coping mechanisms discussed here were chosen for their accessibility, utility, and efficiency. The Table 1 below illustrates these strategies.

**Table 1:** Strategies to combat the deleterious effects of emotional labor on attorney wellbeing.

Mindfulness	The benefits of meditation include improving brain function, reducing anxiety, lessening the attention blink, and increasing flow
Distancing	Journaling facilitates linguistic distancing, which in turn leads to successful emotional regulation
Learning a New Skill/Taking Up a Hobby	Learning a new skill can not only increase competency, but reduce stress hormones and blood pressure, and increase synaptic plasticity

## The lawyer as emotional laborer

The concept of emotional labor was first coined and developed by Arlee Hoschild in her book, “The Managed Heart” (Hoschild, 1983). Emotional labor is based on the concept of emotional dissonance, which is an existing incongruity between the feeling that one is feeling and one that must be expressed in a given situation. Hoschild (1983) argues that different coping mechanisms could attenuate the effect that emotional dissonance has on the psyche of the person experiencing it. She discusses deep acting and surface acting. Deep acting is almost internalizing the feeling that needs to be expressed, to minimize the emotional dissonance. The shortcoming of deep acting would be losing one's sense of self. Surface acting is simply acting as if we are experiencing the desired feeling, at the risk of a developing of a sense of phoniness. Hoschild (1983) studied stewardesses and determined that people who have frequent access to the public and who experience emotional dissonance, are emotional laborers (Hoschild, 1983, p. 10). This category included stewardesses and collection agents, but Hoschild also briefly touched on the possibility of lawyers being emotional laborers (Hoschild, 1983, p. 153). This idea was later expanded by one of Hoschild's students, Jennifer L. Pierce, in her book, “Gender Trials” (Pierce, 1996). Pierce (1996) began the book with the assumption that it was generally accepted that lawyers are emotional laborers. From there, she used the concept to illustrate how lawyers, as emotional laborers, are engendered in their roles because of the deeply masculine nature of the profession, a topic which would require its own analysis and which, although no less important, will not be explored herein.

An in-depth analysis of the “lawyer as emotional laborer” was performed by Yakren (2008), in her article by the same name, where she concludes that a lawyer “must strain to exhibit the requisite outward expressions despite her personal feelings, and, in some cases, will suffer long-term psychological damage as a result (Yakren, 2008). However, while having a sound conclusion, Yakren reaches it by analyzing the conflict between the lawyer's moral responsibility and her ethical obligation to zealously advocate for her client. In a parallel discussion, this article attempts to discuss the conflict between a lawyer's zealously advocating for her client, while internalizing the theory of the case, with her outward externalization of politeness and general amiability toward her adversary, the Court, and even clients. I believe this conflict can be experienced separately or in tandem with the one discussed by Yakren (2008). Further, while Yakren's (2008) theory assumes the existence of a conflict between the lawyer's moral obligation and her ethical obligation to her client, this article attempts to bring to light a conflict that may exist even when the two interests are congruent, such as in the case where the lawyer's case is both morally and ethically on the same page, but the conflict is situationally specific (i.e., on the record v. off the record). Yakren (2008) does address this conflict in her article referring to the conceptualization of the conflict between the personal and professional self, while also stating that “additional conceptualizations of how lawyers perform emotional labor are possible, because it is endemic to the very principles of the legal profession” (Yakren, 2008, p. 157).

While Yakren's conceptualization of emotional labor involves a discussion of “faking in good faith” and “faking in bad faith,” it assumes the truth of only one feeling on the part of the lawyer. The discussion becomes more complicated when both the internal feeling of a lawyer, as well as the one sought to be expressed, are a true representation of the lawyer. While stating that “over time, deep acting may foster a sense of authenticity,” questions persist: Which is the authentic self? What results when both expressions are of the authentic self? If both emotions that the lawyer experiences are true, choosing to deeply act one as opposed to the other, would essentially deepen the conflict between them, a shortcoming that Yakren may be aware of Morris and Feldman (1997) and Yakren (2008), p. 1571.^1^

It is well accepted that “emotional dissonance may be a stressor with deleterious effects. Including ‘personal fragmentation of self,' emotional exhaustion, job dissatisfaction, and ‘personal and work-related maladjustment, such as poor self-esteem, depression, cynicism, and alienation from work”' (Yakren, 2008, p. 163). And while Birke (2010–2011) argues that “lawyers stand to improve their ability to make more reflective decisions that better serve client goals, to understand what may be going on in the minds of negotiators on the other side of the table and of their own clients, and to better understand the interactions between parties,” this article premises that an understanding of the brain's physical and chemical makeup regarding emotional regulation, would stand to benefit lawyers on a personal level (Birke, 2010–2011).

The legal profession is a paradox not only on an individual level, but at a macro analysis. While attorneys attempt to insulate judges and courtrooms of raw emotions and communicate in the most dispassionate of ways, they themselves are managing both their own and their clients' emotions (Spain and Ritchie, 2017). There is not only dissonance between the role of the attorney and his/her conscience, but neurological studies document activation of specific emotion-related regions of the brain when engaging in moral decision-making, arguably involved to some level in the field of law (Spain and Ritchie, 2017). And, while Justice Sotomayor said at her Confirmation Hearing that judges are to “recognize those feelings and put them aside,” and the general consensus positively links emotional repression with rational competence, the pragmatics of practicing law is all about playing on the emotions of a jury, whether it is by “rambo litigants” expressing righteous anger, strategic friendliness when cross-examining an adverse witness, or being strategically aggressive or friendly with opposing counsel (Spain and Ritchie, 2017). To add to this already complicated reality of the profession, recent studies show that attorneys experience vicarious trauma, affecting both their brain and bodies, due to the very nature of their work (Zwisohn et al., 2019). These findings only work to weight down the already emotionally labored attorney.

It is no wonder then that attorneys are 3.6 times more depressed than the general population and are dubbed “professional pessimists” (Sherman, 2015). Additionally, studies find that surface acting as a result of emotional labor was reasonably related to heavy drinking and predicted drinking after work, which is consistent with statistics finding that, although 6.7% of the U.S. population suffers from depression, 45% of attorneys experience depression during their careers; and as much as 21 to 36% of lawyers and others in the legal profession were problem drinkers (Grandey et al., 2019; Henson, 2022). Interestingly, Sherman (2015) contemplates combating emotional labor with a more contemplative practice, to avoid the psychological tension which is a product of the recognized cognitive dissonance within the legal profession (Sherman, 2015).

## Neurobiological considerations

Research has shown that emotions are highly contagious – “Both the quantity and quality of our social relationships affect our mental health, or health behavior, our physical health and mortality risk” (Ioffik, 2017, p. 34). An awareness of our emotional state and the manner in which emotions are expressed can foster improvement in health (Ioffik, 2017). Early understanding of the brain's role in emotional regulation “speculates that the prefrontal cortex modulates the emotional activity of the amygdala,” concluding that people with high prefrontal cortex activity could more easily shut off negative emotions once it is turned on (Mlot, 1998, p. 1005). This is largely based on Daniel Goleman's concept of emotional hijacking, where the amygdala is much quicker than the cortex in responding to stressful situations (Goleman, 1995; Kunnanatt, 2012, p. 53).^2^

Subsequent research, however, has found that “[h]igh levels of activity in the prefrontal cortex, a brain area heavily implicated in self-control, during an emotion regulation tasks have been shown to predict a steep cortisol slope over the course of a day” (Urry et al., 2006, p. 4415; Liberzon et al., 2007, p. 1250; Cunningham-Bussel et al., 2009, p. 694). In fact, the activity of the prefrontal cortex “may dampen amygdala reactivity to emotive stimuli and in this way prevent high levels of emotional reactivity and the associated cortisol output” (Daly et al., 2014, p. 81). This is positive proof that emotional intelligence can assist in emotional regulation by assisting the prefrontal cortex to bypass the amygdala emotional reaction, and therefore reduce stress that may be experienced by lawyers.

Further, emotional intelligence can assist in improving the ability to appropriately regulate and appraise the emotional waive,^3^ which would reduce the frequency of various stress-related disorders (Go et al., 2016, p. 1). Studies have shown that increased cortisol levels are inversely related to cognitive flexibility, therefore it would be expected that training in self-awareness and emotional intelligence, which are shown to decrease cortisol levels, would function in increasing cognitive flexibility. This, in turn, would increase a person's ability to rationalize, and therefore use the cortex when faced with emotional dissonance, or perhaps rationalize the situation and minimize emotional dissonance in the first place (Baruah and Reddy, 2018, p. 2).

Emotional dissonance has been found to be significantly associated with exhaustion, mental distress, and sickness absence (Indregard et al., 2018, p. 592). Additionally, depression and anxiety enhance the production of proinflammatory cytokines, as do physical and psychological stressors (Kiecolt-Glaser et al., 2002, p. 83). Further, negative emotions contribute to overproduction of proinflammatory cytokine (substance secreted at cell level associated with pathological pain) (Kiecolt-Glaser et al., 2002). This can be oversimplified by the understanding that negative emotions affect the immune system (Kiecolt-Glaser et al., 2002). Similarly, emotions in general, affect the endocrine system (Kiecolt-Glaser et al., 2002). Importantly, “intense emotional experiences have the capacity to permanently alter neuroendocrine and autonomic responses” (Kiecolt-Glaser et al., 2002).

## Emotional intelligence and affective neuroscience

Some research has been done regarding the protective role of emotional intelligence against the emotional dissonance present in emotional labor (Jordan et al., 2002, p. 1; Mikolajczak et al., 2007, p. 1107). Salovey and Mayer (1990) defined emotional intelligence as “the ability to monitor one's own and other's feelings and emotions, to discriminate among them, and to use this information to guide one's thinking and action” (Salovey and Mayer, 1990, p. 189). It has been proposed that emotional intelligence and emotional labor should be studied together as persons in intensely emotionally laborious jobs would benefit from emotional intelligence (Grandey, 2000, p. 95; Opengart, 2005, p. 3). And, in a prodigious paper, Farah Naqvi, proposes that people with “high emotional intelligence will have better ability to deal with emotional labor” (Naqvi, 2009, p. 27). I venture to extend her proposition and hold that self-awareness, which is necessary in emotional intelligence can be used to combat emotional dissonance inherently present in emotional labor by either preventing it from occurring or addressing it and reappraising it when it does occur.

In the words of Hagreen (2004), “[p]erhaps the answer lies with the individual”. Closer to this article's premise still, Amiram Elwork, director of the Law-Psychology Graduate Training Program at Widener University and the author of Stress Management for Lawyers, suggested that lawyers can reduce stress by becoming emotionally intelligent (Hagreen, 2004, p. 3). Further, in Brown (2012), boldly asserted that lawyers “with ‘emotional intelligence' complementing their technical legal knowledge are often better able to exercise the professional judgment that integrates multiple facets of their presenting problems” (Brown, 2012, p. 189).

In, Douglas (2015) did an in-depth review of the positive effects that emotional intelligence could have on the legal profession (Douglas, 2015, p. 56). There is no doubt that emotional intelligence can improve a lawyer's wellbeing from a social theory perspective, by improving self-awareness, self-regulation, motivation, empathy, and social competence (Douglas, 2015). Since the effects of emotional labor can be seen at a neural level, it is anticipated that, conversely, emotional intelligence can and will have a beneficial impact on the brain. Further, it has been argued that emotionally intelligent individuals are able to activate their cortical brain to sense emotions before they occur (Kunnanatt, 2012). This ability can be trained through emotional intelligence development programs.^4^

Emotional intelligence will help lawyers diminish the emotional dissonance they feel in various situations by allowing them to reappraise their emotions. “Cognitive reappraisal is defined as a form of cognitive change that involves a reinterpretation of an emotion-eliciting situation in order to modify its emotional impact” (Megias-Robles et al., 2019). Similarly, “[e]motional Regulation (ER) refers to the overall set of skills and cognitive and behavioral processes used to influence the nature and intensity of the emotion the moment and/or situation one is having this emotion, the reappraisal of the emotion, and the actual display of the emotion” (Van Beveren et al., 2019). While emotional reaction can increase stress and therefore stress hormones, such as cortisol, self-regulation increases resilience, resulting in a shorter stress response, and therefore, a reduced effect of cortisol on the body (Feder et al., 2009).

Emotional intelligence can mediate the negative effects of emotional labor on attorneys. In his recent article, *The High Cost of Emotional Labor*, Alan Morantz recognizes that emotional labor, such as summoning interest in a client's ill-conceived ideas, can feel exhausting and inauthentic (Morantz, 2021). He also posits that “[i]f you're an extrovert or have higher emotional intelligence or confidence in your ability to perform emotional labor, it's a little easier for you psychologically” (Morantz, 2021). However, if you are a person who values authenticity and find emotional regulation disingenuous, you will have a harder time with the psychological demands of emotional labor and might struggle with burnout, depersonalization, lower job satisfaction, higher absenteeism, higher turnover, and lower work engagement (Morantz, 2021). Studies abound which find statistical relationships between emotional intelligence and the burnout that results from emotional labor.^5^

Being emotional intelligent is becoming increasingly advantageous, particularly since studies have shown that non-verbal communication delivers more meaning faster than language and conveys feelings and emotions more accurately (Delmonte, 1991, p. 1). While there are different ways to improve emotional intelligence, neuroscientific findings suggest distancing, reinterpretation, reappraisal, and practicing mindfulness (Marin and Ochsner, 2016, p. 142). Additionally, neuroimaging studies have found that reappraisal is effective at dampening or enhancing responses associated with affective responding (reducing amygdala reactivity). Similarly, these studies confirm the social regulation of emotions that occurs when peers are present. Their presence can influence recruitment of regions of the brain that trigger either positive or negative emotions, consistent with the level of stress or confidence an attorney may feel while presenting his or her case before a Judge in front of his or her peers (Marin and Ochsner, 2016).

## Mindfulness

Meta-awareness of emotions through non-reactive observance facilitates emotional regulation (Compare et al., 2014). Additionally, meditation has been propagated as a beneficial intervention, based in neuroplasticity, when dealing with, what has been subsequently dubbed, as a behavioral anomaly – emotional dissonance (Santhosh and Krishnankutty, 2011, p. 685). In fact, when discussing emotional dissonance, it is said that “the concept of neurogenesis and neuroplasticity is the only ray of hope in bringing emotional harmony” (Santhosh and Krishnankutty, 2011).

In their book, *Altered Traits*, the authors make a good case for why mindfulness can increase physical wellbeing (Goleman and Davidson, 2017). Since trier stress – stress from being judged – is known to increase blood pressure and cortisol levels, it is not surprising that meditation, which makes the individual more immune to emotional hijacking and increases connection between prefrontal cortex and amygdala, can facilitate learned resilience. This is particularly important to lawyers, part of whose job it is to constantly be actually or apparently judged. Even from a pragmatic approach, meditation is said to lessen the attention blink.^6^ Particularly, in our society, where amount of information is thrown at us at inhuman speeds, Nobel laureate Herbert Simon's epiphany that a wealth of information is a poverty of attention, is increasingly poignant.

Meditation is also said to improve “flow.^7^” Given that the stress from being disingenuous can lead to toxic cytokines (Goleman and Davidson, 2017), the fact that mindfulness-based stress reduction (MBSR) reduced the number of toxic cytokines creating inflammation is further proof that meditation can be used to reduce the level of stress that emotional labor puts on lawyers. Some cardiologists also support meditation for lowering blood pressure. Additionally, MBSR can help increase telomers, which measures cell life (Goleman and Davidson, 2017).

Moreover, tentative findings point to meditation for strengthening insula in the brain, a region that powers emotional self-awareness; and singular cortex, a region that powers self-regulation, and slows down brain atrophy and brain aging. Further, because part of being emotionally intelligent is being empathetic and responsive to the emotions of others, meditation improves the emotional intelligence by increasing empathy in meditators (FMRI studies showed empathy increased by 800% in meditators) (Goleman and Davidson, 2017). Meditation also reduces anticipatory anxiety, which is an axiomatic component of an attorney's life (whether anticipating a decision from court, a question for your witness from opposing counsel, or a response to a settlement demand).

If the data was not convincing enough, it was found that a daylong retreat can boost immune system, so taking a day off and just doing things other than working with your brain, may have a general good impact on the immune system (Goleman and Davidson, 2017). To that effect, concentration is said to be improved by hands-on work, a topic that will be more thoroughly discussed *infra*.

## Distancing

Psychological distancing is a form of cognitive reappraisal – perceiving the stimuli with a perceived spatial and temporal distance. It is related to adaptive mental and physical health outcomes. Research suggests that shifting language to be more distant (i.e., linguistic distancing) can have adaptive emotion regulatory effects, such as lower perceived stress and depression symptoms, better general wellbeing, energy and vitality, and better emotional regulation (Shahane and Denny, 2019, p. 200). Neurologically this is also confirmed by temporal and spatial brain imaging studies that suggest emotions are represented in the brain as a set of semantic features and interact with several lexical, semantic and syntactic features in different brain regions (Hinojosa et al., 2019). Notably, Hinojosa et al. (2019) work is a steppingstone in establishing that language and emotion are mutually interdependent. The work supports constructivist theorists, who posit that language, which can undoubtedly be shaped by emotions, not only identifies and communicates emotions, but shapes emotion, perception, and is able to develop emotional competence (Lindquist et al., 2015; Dunabeitia, 2020, p. 868). Consistently, it was found that emotional regulation increased linguistic markers of social and temporal distance. Participants who showed greater linguistic distancing were more successful emotional regulators (Nook et al., 2017, p. 337). In Nook et al. (2017) experiment, writing by using psychological distant language in physical, social and temporal domains, spontaneously reduced negative affect – reducing present tense increased temporal distancing; reducing the use of the work “I” increased physical distancing (Nook et al., 2017).^8^ Tangentially, reappraisal writing functions to decrease anxiety levels and can attenuate negative emotions to some degree, while decreasing alpha asymmetry (Wang et al., 2015).^9^ In essence, cognitive reappraisal – changing the meaning of a stimulus to alter its emotional impact – is an effective emotional regulation technique. A similar effect was found by Nook et al. (2020) after an experiment involving spoken language rather than written (those who used more distancing language were better able to regulate emotions; conversely, good regulators used a larger amount of distancing language) (Nook et al., 2020, p. 525).

To this point, journaling is an important tool to self-regulation and reducing the stress that emotional labor has on an attorney. Brain scans have shown that putting things down on paper reduces activity in the amygdala (Sample, 2009). Further, journaling can increase self-awareness, which in turn can lead to increased job-satisfaction (Thatcher, 2021). Journaling also strengthens the pathways in the brain that are responsible for disbursing the hormones serotonin and dopamine (Wapner, 2008). Clinical studies have also shown that journaling aids the brain in processing trauma (Stice et al., 2007, p. 863; Hasanzadeh et al., 2012, p. 183) (see discussion supra on vicarious trauma experienced by attorneys). Moreover, studies have shown that journaling can reduce cortisol level, which as discussed, is the primary culprit in processing stress reactions to emotional labor, therefore affecting the immune system (vybey, 2022). Moreover, although focused on the effects of COVID-19, a recent study found that psychological distancing, which is inherently involved in journaling, was associated with reduction on perceived stress (Dicker et al., 2022).

A note of caution is warranted in light of Nook et al.'s findings that distancing can be increased by using past tense and decrease usage of the pronouns “I” (*see supra*). When journaling, a conscious effort should be made to heed Nook's advice. Temporal and spatial distancing by using remote language will alleviate the effects of stress on the person journaling. Attorneys should be no stranger to this technique, since a part of persuasive writing which is learned in introductory law school classes, is to write in such a way to distance your client from the wrongdoing, if you are representing the Defendant. Similarly, an law school student is taught to outline the direct impact of the wrongdoing on his/her client or the victim by using primacy and recency, and active voice, if they are representing Plaintiff or prosecuting a crime, by appealing to – no surprise – the emotional side of the judge and/or jury.

## Learning a new skill/taking up a hobby

Finally, an effective strategy to combat the deleterious effects of emotional labor, is taking up a new skill or hobby. Ever wonder why hobbies are listed on a resume or discussed in an interview? Aside from having the potential to appraise future employees of an attorney's conscientiousness or personality, research supports the theory that individuals who take on a new skill or a hobby demonstrate an ability to de-stress. For example, it is commonly accepted that learning something new relieves stress by increasing competency (Zhang et al., 2018). Physiologically, learning a new skill thickens the prefrontal cortex and builds new pathways within the brain, which are involved in logical thinking and, as seen above, can combat the effects of emotional labor (www.piedmont.org) (The Mind-Body Benefit of Learning a New Skill, 2023). Additionally, if the new skill involves physical activity, it can increase blood flow and reduce blood pressure, as well as stress hormones. In fact, studies show that physical activity increases a brain's stress resilience by increasing synaptic plasticity and reducing inflammatory factors (Nowacka-Chmieliewska et al., 2022). Not to be overshadowed, studies have shown that making art can also reduce stress hormones, including cortisol, in the body (Otto, 2016).

To this point, it is not uncommon to see lawyers taking up mycology (the study of mushrooms), baking, motor racing, tattoo art, or cycling, to name a few (King, 2016). Anecdotally, growing greens can reduce mental stress and minimize the risk of burnout (Zeremba, 2022). In fact, scientific investigations support this theory by discussing how some microbes in soil are important to human health (Steffan et al., 2017).

## Conclusion

Whatever type of law you practice, it is important to understand that, as attorneys, we are emotional laborers and we suffer the effects that emotional labor has on our psyche (brain, emotional health, physiology). Our profession inherently involves emotional dissonance in the incongruity present in our interactions with everyone in our professional lives, from a client, to an adversary, a Judge, and everyone in between. By the very nature of our work as problem solvers, we often mediate our own feelings in a given situation depending on the actors involved. In the deposition which prompted my interest in this topic, my feelings toward the attorney representing the Defendant in that case were at once inimical and amicable, depending on whether the stenographer's machine was turned on or off. Understanding the effects that emotional labor has on attorney's stress level and neuro-health can help improve not only an attorney's wellbeing, but his/her job satisfaction, and reduce the rates of suicide, alcoholism, depression, and drug addiction associated with the legal community. Simple but intentional methods of reducing the effects of emotional labor can be implemented. To that effect, emotional intelligence will aid an attorney in identifying emotional laborious situations and reducing their impact before they present within the attorney through increased cortisol levels or a weakened immune system. Practicing mindfulness and self-awareness are key to improving emotional intelligence. Additionally, it is important that attorneys learn to psychologically distance themselves from emotionally intense situations by journaling in a passive, dissociated voice, as well as picking up new hobbies and learning new skills, whether physically demanding or not, to reduce their cortisol levels and strengthen their prefrontal cortex.

Wellbeing is ultimately an ethical issue which attorneys have a principled obligation to abide by. The first Model Rule of Professional Conduct states that a lawyer shall provide competent representation to a client, meaning poses the competence, knowledge, thoroughness, and preparation reasonably necessary for client representation. Further, attorneys need to maintain a number of continuing legal credits for their license yearly. Several of these credits must be in ethics and professional responsibility. Competence and ethics are intrinsically tied with an attorney's wellbeing. Bar Associations have, in recent years, formed committees on attorney wellbeing, with a focus on outreaching to formerly disciplined attorneys in hopes of rehabilitating them. Additionally, continuing legal education courses dealing with self-compassion, distressed lawyers, managing stress, or avoiding compassion fatigue – alluding to the emotionally laborious role of attorneys – often offer ethics credits. And while these efforts are an appropriate response to many issues arising out of an attorney's inability to handle the emotional dissonance associated with the profession, an interdisciplinary approach is recommended. Collaboration with psychologists, neurobiologists, and affective neuroscientists is encouraged for the education of attorneys on the deleterious effects that emotional labor has on their wellbeing, as well as the advancement of emotional intelligence development training programs.

With the amount of research that exists not only in legal academia, but also neuroplasticity, neurolinguistics, and psychology, it is ironic then that emotional labor is “a dimension of work that is seldom recognized, rarely honored, and almost never taken into account … as a source of on-the-job-stress” (Hoschild, 1983, p. 153). The people most affected by emotional labor appear to be the ones least aware of the problem and its underlying mechanisms. It would be fitting for the local, state, and national bar associations proclaiming to offer services to enhance the skills of their members, to also offer meaningful training services intended for their members' wellbeing. To that effect, developing programs to educate and bring awareness to the issue, and working to provide training and opportunities for attorneys to alleviate their professional stress, is encouraged.

## Author contributions

The author confirms being the sole contributor of this work and has approved it for publication.

## Footnotes

^1^It is noted that not only Yakren, but the majority of the body of research focuses on emotional dissonance being experienced as a result of having to express an emotion that one may not actually feel. However, this article is premised on an analysis of two feelings that an attorney may simultaneously feel, which are both true, but which are incongruent with each other.

^2^Goleman also suggested here that mindfulness may combat low grade anxiety.

^3^This waive involves the nonadrenal system in the brain, which is considered to play an important role in emotion and stress.

^4^The training usually involves emotional mapping (helps individuals to understand their biological inclinations; diagnosing emotional (interrupting a learned, established neural pathway; emotional authentication (determining how productive or unproductive emotional responses are); emotional navigation (exercises designed to consciously slow down and regulate emotional reactions); empathy building; and influence building (depends on proficiency in skills such as communication, assertiveness, negotiation, and conflict management).

^5^Guy and Lee (2013), p. 261 (finding that one's ability to regulate one's own emotions decreases burnout); Mangi et al. (2012), p. 469 (finding that emotional labor has significant impact on work engagement and cognitive dissonance has significant negative effect on work engagement, while emotional intelligence mediates and reduces the negative relations between cognitive dissonance and work engagement); Abraham (1999), p. 441 (finding that emotional dissonance aroused feelings of job dissatisfaction in high self-monitors and social support lessened the negative impact of emotional dissonance on organizational commitment); Lee et al. (2018).

^6^Attention blink refers to the blind spot in memory where a thought approaches your consciousness, then disappears before one has had time to fully grasp it.

^7^Flow refers to the feeling one gets when one feels like they are “in the zone,” time dilates while one is researching, writing, or performing a taxing project like difficult math or physical activity.

^8^For a lay overview of how grammar can improve mindfulness, see Weinstein (2020). In it, Weinstein superbly discusses removing ourselves from the agency of an undesired action by using passive voice and its psychotherapeutic benefits; the importance of journaling to learn from our mistakes; and many other grammatical life-hacks. See also, Pennebaker (2011).

^9^Alpha asymmetry is believed to measure propensity to adopt approaching or withdrawing behavior.
